# Prevalence and Quantification of the Effects of Sexual Harassment Victimization of School-Aged Adolescents

**DOI:** 10.3390/children11010023

**Published:** 2023-12-24

**Authors:** Verónica Marcos, Dolores Seijo, Álvaro Montes, Ramón Arce

**Affiliations:** Forensic Psychology Unit, Faculty of Psychology, University of Santiago de Compostela, 15782 Santiago de Compostela, Spain; veronica.marcos.martinez@usc.es (V.M.); mariadolores.seijo@usc.es (D.S.); alvaro.montes.campos@usc.es (Á.M.)

**Keywords:** mental health, adolescence, educational field, violence prevention, gender

## Abstract

Background/aim: Sexual harassment has become a serious social and public health problem in adolescents, causing adverse effects on mental health. Nevertheless, some behaviours arise that, due to their characteristics, might be misinterpreted as sexual harassment. A field study using a survey with non-probabilistic accidental sampling was designed in order to estimate the prevalence of sexual harassment victimization in the Spanish adolescent population as well as to quantify the harms. Method: A total of 1028 Spanish adolescents, 54.3% females and 45.7% males aged 13–17 years (*M* = 15.21, *SD* = 1.03), responded to a diagnostic measure of sexual harassment victimization and an inventory measure of internalizing and externalizing mental health problems (MHPs). Results: The results showed a significant prevalence of diagnosed sexual harassment victimization of school-aged adolescents, 24.1%, 95% CI [0.215, 0.267], with adverse effects on internalizing and externalizing MHPs. As for the internalizing MHPs, the results exhibited moderate adverse effects on depression, anxiety, somatic burns, posttraumatic symptoms, and obsessive–compulsive symptoms, as well as mild adverse effects on social anxiety. Regarding externalizing MHPs, the results revealed moderate adverse effects on hyperactivity–impulsivity, anger control, and antisocial behaviour, as well as mild adverse effects on attention problems, aggression, and defiant behaviour. In addition, it was confirmed that sexual harassment victimization affects adolescent females to a greater extent, with the effect being significantly greater in internalizing than in externalizing MHPs. Conclusions: The results obtained are discussed and future lines of research and intervention are proposed to promote the implementation of prevention and intervention programs that address this phenomenon and, in turn, improve the physical, psychological, and social well-being of adolescents.

## 1. Introduction

Sexual violence and sexual harassment have become social and public health problems of great concern [1], especially when they begin in childhood and/or adolescence [2,3,4]. In this regard, in their recent research with a sample of 13,052 US children and adolescents, Gewirtz-Meydan and Finkelhor [5] found that the majority of sexual harassment victimization is committed by other children or adolescents, most often by adolescents aged 14–17 years, and mainly by acquaintances. In terms of gender, epidemiological studies have consistently confirmed a prevalence of unwanted sexual behaviour in females, both in the physical context—face to face—[6,7,8,9] and non-conclusive results in the virtual context [10,11,12]. It is noteworthy that a significant prevalence of sexual harassment victimization has also been observed among adolescent males [4,5].

The most widespread definition of sexual harassment at school was provided by the American Association of University Women [13], which defines it as a set of unwanted sexual behaviours that interfere with the lives of young people. Similarly, the American Psychological Association [14] refers to sexual harassment as unwelcome offensive conduct of a sexual nature. Both definitions agree that the sexual nature of the conduct is unwanted. Nevertheless, these are not operational, nor does it delimit (differential diagnosis) child and adolescent sexual harassment from other behaviours within the relational framework of adolescents that begin in this period of development (e.g., kissing, touching, and flirting), and which, due to their characteristics, may be erroneously interpreted similarly to situations of sexual harassment or from other criminal typologies, such as sexual abuse and aggression.

As for relational behaviours between children and adolescents growing into sexual harassment, three criteria (bullying criteria) must be met for these behaviours: intentionality of the behaviour/action, periodicity, and chronicity [15,16]. Thus, relational behaviours between children and adolescents that do not meet those criteria are not sexual harassment. A differential diagnosis from other sexual nature crimes implies a strict differentiation between sexual abuse and sexual violence. Sexual abuse for school-aged children and adolescents under the age of consent is referred to as some form of sexual behaviour or activity between adults and those underage to consent [17]. Sexual violence is some forced (using coercion or violence) sexual act (e.g., rape, sexual assault) or an attempt at a sexual act [18].

Consequently, for a proper diagnosis of sexual harassment victimization of school-aged children and adolescents, it is necessary to specify the behaviours and strategies of sexual harassment, as is the case with other manifestations of bullying (e.g., bullying victimization). Harassment victimization in school-aged adolescents consists of being a target of intentional, frequent, and chronic unwanted sexual behaviours or strategies.

Sexual crime victimization, as a criminal action, is associated with harm to the victim which, as a psychological injury, is of an emotional or mental character [19]. The scientific literature refers to this harm as having adverse effects on mental health and cognition [6,7,8,9,10,11,12,20,21,22,23]. Given the age of the victims (childhood and adolescence), such mental health effects manifest themselves in both internalizing and externalizing symptoms. In the domain of internalizing mental health problems (MHPs), depression [11,21,23], anxiety [11,20,21,23], and suicidal ideation were registered as primary diagnoses [3,24], both in face-to-face—offline— [25,26] and virtual contexts—online [27].

Adverse effects were also observed in externalizing MHPs, specifically criminal and antisocial behaviour [8,28,29]. In this sense, following a meta-analytic review [30], the likelihood of antisocial behaviours among victimized adolescents was quantified to be almost twice as high as among their non-victimized peers.

Based on this review, a field study (a survey) was designed to find out the prevalence of sexual harassment victimization in school-aged adolescents, as well as the adverse effects and quantification of the magnitude of internalizing and externalizing MHPs of sexual harassment victimization and the interaction with gender.

## 2. Method

### 2.1. Participants

A total of 1028 Spanish adolescents participated in this study, with 54.3% females (*n* = 558) and 45.7% males (*n* = 470) aged between 13 and 17 years old (*M* = 15.21, *SD* = 1.03). Regarding the academic year, 36.3% were in their 3rd year of compulsory education (14–15 years), 39.0% were in their 4th year of compulsory education (15–16 years), 17.6% were in the 1st of their Baccalaureate (16–17 years), 6.0% were in the 2nd year of their Baccalaureate (17–18 years), and the remaining 1.1% were in their formative cycles. Regarding the type of secondary school, 73.5% were in a public school, 20.8% were in a state-subsidized school, and 5.6% were in a private school.

### 2.2. Design and Procedure

A non-probabilistic convenience sampling survey was designed (confidence level: 95%; margin of error ±3.03%) to estimate the prevalence of sexual harassment victimization in the Spanish adolescent population, as well as to quantify the damages. In order to obtain the sample, first, a request was sent to the schools. Then public and private Galician (northwest of Spain) schools were randomly selected from the administration repository of schools and were contacted. Five schools responded affirmatively to participating in this study (four public and one private). Once the request was accepted, informed consent was obtained from the parents or legal guardians (mandatory for <16 years).

After giving informed consent, the participants (all those who responded to the call) filled in the questionnaires in classes, responding voluntarily, anonymously, and individually, while being supervised by professional staff. The tests were administered to participants during school attendance. The order of obtaining the measurements followed a standard rotation procedure [31] to counterbalance a possible interaction effect among the variables. The collection, storage, and treatment of the data were carried out according to the Spanish Data Protection Act [32].

### 2.3. Measure Instruments

An ad hoc questionnaire was designed to obtain socio-demographic information (i.e., gender, age, academic year, and type of school) that was self-reported by the participants.

A diagnosis of harassment requires not only that the person has been subjected to harassing behaviours, in this case, sexual harassment, but also that certain criteria must be met to discriminate sexual harassment from other types of actions against sexual freedom (differential diagnosis): intentionality of the conduct, periodicity, and chronicity [16,33].

A differential diagnosis involves discrimination from other crimes against sexual freedom, i.e., sexual abuse and sexual assault. Abuse occurs when the victim is under the age of consent and the perpetrator is over the legal age of consent. Therefore, sexual harassment of school-aged children and adolescents must be performed by peers (perpetrator [34]); otherwise, it would be abuse (some of the literature has equated child abuse with aggression). Aggression, on the other hand, involves the use of force, intimidation, or coercion. Substance use is abuse or aggression, according to the applicable literature, but not applicable to bullying.

As a measure of behaviours or strategies that constitute sexual harassment at school, a context effect was found in the measurement instruments: traditional bullying and online bullying. Thus, surveys were found for the measurement of traditional bullying behaviours [13,35,36] and psychometric instruments of online bullying [37,38]. It was pointed out that the instruments introduced measures that implied the use of violence or force, intimidation, or coercion as aggression (e.g., someone has forced you to kiss him/her) or wording that did not directly imply an intention to harass (intentionality criterion).

The measures of sexual harassment behaviours and strategies adapted to one or the other context were collated. These measures, which are the basis of the literature reviewed, were of limited validity (they only measure one context or the other and thus, partially assess the construct), without discrimination of other analogous constructs (differential diagnosis), and with diagnostic error (sexual harassment is de facto diagnosed without verifying intentionality, frequency, or chronicity). Some items did not measure (invalid) harassment (e.g., has any student kissed or hugged you or tried to?). Consequently, a pool of items was constructed based on the instruments found, combining, where it was possible, the use of the behaviour or strategy in both contexts in the same item, i.e., the items were reworded to imply that the bullying behaviour/strategy was not an aggression and was intentional.

Taking into account the resulting set of items and the corrected item–test correlation calculated, those behaviours or strategies with a correlation (*r*) < 0.40 were eliminated, such that they were not measuring the same construct. This resulted in a measure of harassment consisting of 19 sexual harassment behaviours/strategies, to which participants responded on a 5-point Likert-type scale for frequency (1 = *never or rarely happens to me*; 2 = *once a month*; 3 = *two or three times a month*; 4 = *once a week*; 5 = *several times a week*).

In the case of a positive response and frequency greater than two or three times a month or more, participants were asked about the periodicity (diagnostic criterion of chronicity of bullying) with which they were being or had been subjected to this bullying behaviour or strategy: “up to one month”, “up to three months”, “up to six months”, or “up to one year or more”. For a diagnosis of sexual harassment victimization, participants were required to have been subjected to at least one sexual harassment behaviour, weekly or more frequently (periodicity criterion), and for longer than 6 months (chronicity criterion [15,16]). From the participants in this study, the resulting inventory of sexual harassment behaviours or strategies (see Appendix A) presented reliability (internal consistency) sufficient for measures in applied contexts that serve to make important decisions (e.g., diagnosis), *α* = 0.90 [39].

As for the assessment of psychological adjustment, the *Sistema de Evaluación de Niños y Adolescentes* (Assessment System for Children and Adolescents) [40] was administered. This scale consists of 188 items, structured into 3 measures: mental health problems, vulnerability, and personal resources. The response scale is a 5-point Likert-type scale: *never* (1), *rarely* (2), *sometimes* (3), *often* (4), and *always* (5). Within this study, the measurement of mental health problems (MHPs) was used: internalizing problems (i.e., depression, anxiety—generalized, social anxiety, somatic complaints, and obsessive–compulsive) and externalizing problems (i.e., attention problems, hyperactivity–impulsivity, anger control, aggression, defiant behaviour, antisocial behaviour). In the present study, the internal consistency and Cronbach’s alpha for internalizing and externalizing MHPs were 0.89 and 0.91, respectively.

### 2.4. Data Analysis

The prevalence of sexual harassment victimization was calculated by obtaining the zeta value for the difference between the observed probability with a constant, 0.05, effect or trivial prevalence [41]; obtaining the effect with Cohen’s *h* and interpreting this qualitatively as small (*h* = 0.20), moderate (*h* = 0.50), large (*h* = 0.80), or more than large (*h* = 1.20) [42,43]; and quantifying the magnitude of the effect with the Effect Incremental Index (EII [44]).

A MANOVA test was run for the comparison of means using a customized design with the victimization factor (victimized vs. non-victimized) and the interaction between the victimization factor and gender (females vs. males), given that the literature has shown that females and adolescent victims of sexual harassment present greater harm in internalizing MHPs than males and adolescent victims [45]. The effects of the frequency of harassment actions were not controlled (covariate) in the design because it is a criterion for diagnosing sexual harassment. Similarly, due to its effect on internalizing or externalizing mental health problems, age was not included as a covariate in the design (participants were in a shift period from externalizing to internalizing mental health problems, thus confusing the effects). In multivariate contrasts, a multivariate Pillai–Bartlett trace test was conducted since it is robust to a homogeneous variance–covariance assumption [46]. The heterogeneity of variance was also observed in univariate comparisons (Levene’s test), which may cause deviations in the significance of the results [47]. To deal with this contingency, the value of the theoretical *F* (Box’s test for the equality of covariance matrices) was contrasted with the empirical *F* to validate the correct acceptance or rejection of the null hypothesis: if the empirical *F* was higher than the theoretical *F*, then the alternative hypothesis was correctly accepted, and vice versa [48]. This criterion was met for significant univariate *F* values.

In multivariate contrasts, the effect size was calculated as $\eta_{p}^{2}$ and the standardised mean difference with Hedges’ unbiased *g*, the latter being for the comparison of adolescent victims in the significant interaction between the factor’s victimization and gender. The magnitude of effect sizes was interpreted qualitatively by taking Cohen’s categories [43] of large (*g* ≥ 0.80, $\eta_{p}^{2}$ ≥ 0.1379), moderate (*g* = 0.50, $\eta_{p}^{2}$ = 0.0588), and small (*g* = 0.20, $\eta_{p}^{2}$ = 0.0099) and using the Probability of Superiority of Effect Size (PS_ES_ [44]); that is, the percentage of effect sizes out of the total that would exceed the observed one, and the variance explained for $\eta_{p}^{2}$.

The model error was computed with the Probability of an Inferiority Score (PIS [49]). A derivation of the BESD was used to quantify the deficits resulting from victimization [50].

Moreover, the reliability (internal consistency) of the measurement instruments was calculated in the sample of the present study.

## 3. Results

### 3.1. Prevalence of Sexual Harassment

Nearly one-fourth, 24.1% (*n* = 248), 95% CI [0.215, 0.267], of the adolescents were diagnosed (reliability, *α* = 0.90) with sexual harassment victimization, which was a significant prevalence ((>0. 05), *Z* = 28.10, *p* < 0.001), with a more than large effect size (*h* = 1.45, 95% CI [1.42, 1.48]), and greater than 84.85% (PS_ES_ = 0.8485) of all possible sizes. The increase in (incremental effect: prevalence over a trivial effect) sexual harassment was 79.2%, EII = 0.792. Concerning gender, female adolescents (30.1%) were significantly more (χ^2^(1, *N* = 1028) = 23.87, *p* < 0.001) victimized than male adolescents (17.0%), although the effect size was small (Prevalence Ratio = 1.77) and larger than 58.71% (PS_ES_ = 0.5871).

### 3.2. Effects of Sexual Harassment Victimization on Internalizing MHPs

The results exhibited a significant multivariate effect (*F*(6, 1019) = 16.36, *p* < 0.001), with a full power of 1 − β = 1.00 (i.e., the probability of incorrectly rejecting the null hypothesis was 0, and β was lower than α), of the sexual harassment victimization factor in internalizing MHPs, explaining 8.8% $(\eta_{p}^{2}$ = 0.088, 95% CI [0.053, 0.117]) of the variance. Consequently, victims of sexual harassment differed in internalizing MHPs. Similarly, the interaction between sexual harassment victimization and gender was also significant (*F*(12, 2040) = 19.76, *p* < 0.001) with a total power of 1 − β = 1.00 (the probability of a false rejection of the null hypothesis is 0, and β is lower than α) and accounting for 10.4% ($\eta_{p}^{2}$ = 0.104, 95% CI [0.076, 0.124]) of the variance. That is, female and male adolescent victims and non-victims differed in internalizing MHPs.

Regarding the univariate effects (see Table 1), the results showed that victims of sexual harassment reported significantly more symptoms with a moderate to large effect size (0.50 < *g* < 0.80) that was greater than 68.79% of all possible effects in depression, 67.72% in anxiety, and 70.54% in posttraumatic symptoms than non-victims. They also reported a moderate effect size (g ≈ 0.50) in somatic complaints, which was greater than 65.91%, and in obsessive–compulsive symptoms, which was greater than 59.87%.

Quantitatively, victims of sexual harassment reported 33.0% (*r* = 0.330) more depressive symptoms than non-victims; 30.9% (*r* = 0.309) more anxiety symptoms; 8.5% (*r* = 0.085) more social anxiety symptoms than non-victims; 27.9% (*r* = 0.279) more somatic complaints symptoms than non-victims; 35.9% (*r* = 0.359) more posttraumatic symptoms than non-victims; and 24.3% (*r* = 0.243) more obsessive–compulsive symptoms than non-victims. Notwithstanding, the model error (probability of the victim group scoring below the non-victim group mean) was 24.2% for depression, 25.8% for anxiety, 43.3% for social anxiety, 28.1% for somatic complaints, 22.1% for posttraumatic symptoms, and 30.8% for obsessive–compulsive symptoms.

The univariate effects of the interaction between victimization and gender (see Table 2) revealed a significant effect on depression, anxiety, social anxiety, somatic complaints, posttraumatic symptoms, and obsessive–compulsive symptoms. The standardized mean difference between female (*n* = 168) and male (*n* = 80) victims of sexual harassment was significant (lower bound of the 95% CI > 0.20) and of a large magnitude (*g* > 0.80) with greater than 77.34% of all possible effects on anxiety; it was of a moderate to large magnitude (0.50 < *g* < 0.80) in depression, somatic complaints, posttraumatic symptoms, and obsessive–compulsive symptoms with effect sizes greater than 67.00%, 67.72%, 68.02%, and 65.17%, respectively; and it was of a small to moderate magnitude (0.20 < *g* < 0.50) in social anxiety, with a size greater than 60.64%.

Quantitatively, female adolescent victims of sexual harassment reported 29.6% (*r* = 0.296) more depressive symptoms; 46.8% more anxious symptoms (*r* = 0.468); 18.7% (*r* = 0.187) more social anxiety symptoms; 30.9% (*r* = 0.309) more somatic complaints; 31.3% (*r* = 0.313) more posttraumatic symptoms; and 26.5% (*r* = 0.265) more obsessive–compulsive symptoms than male adolescent victims. Nevertheless, the model error (probability of the victim group scoring below the mean of the non-victim group) was 26.84% for depression, 14.5% for anxiety, 35.2% for social anxiety, 25.8% for somatic complaints, 25.5% for posttraumatic symptoms, and 29.1% for obsessive–compulsive symptoms.

### 3.3. Effects of Sexual Harassment Victimization on Externalizing MHPs

The results exhibited a significant multivariate effect (*F*(6, 1019) = 19.84, *p* < 0.001), with a total power of 1 − β = 1.00 (the probability of incorrectly rejecting the null hypothesis is 0, and β is lower than α), of the sexual harassment victimization factor in externalizing MHPs, explaining 10.5% ($\eta_{p}^{2}$ = 0.105, 95% CI [0.068, 0.136]) of the variance. Thus, adolescent victims and non-victims of sexual harassment differ in the externalizing symptomatology developed. Similarly, the interaction between sexual harassment victimization and gender was also significant (*F*(12, 2040) = 6.96, *p* < 0.001) with a total power of 1 − β = 1.00 (the probability of incorrectly rejecting the null hypothesis was 0, and β was lower than α) and accounting for 3.9% ($\eta_{p}^{2}$ = 0.039, 95% CI [0.020, 0.052]) of the variance. That is, adolescent victims and non-victims differ in externalizing MHPs. Nevertheless, the effect was significantly larger for internalizing MHPs (the confidence interval was larger) than for externalizing MHPs.

For the victimization factor, the univariate effects (see Table 3) revealed that victims of sexual harassment reported significantly more symptoms with a moderate to large effect size (LL 0.50 < *g* < UL 0.80) and greater than 67.36% of all possible effects on anger control and greater than 65.54% on antisocial behaviour than non-victims. The univariate effects also reported a moderate effect size (95% CI of *g* is greater than 0.50) greater than 64.80% on hyperactivity–impulsivity, and moderate effect size and greater than 62.55% on defiant behaviour. Finally, univariate effects reported a small to moderate effect size (LL 0.20 < *g* < UL 0.50) on attention problems and aggression, being greater than 62.16% and 61.41% of all possible effects on attention to problems an aggression, respectively. 

Quantitatively, the victims of sexual harassment reported 21.5% (*r* = 0.215) more attention problems; 26.1% (*r* = 0.261) more manifestations of hyperactivity–impulsivity; 30.5% (*r* = 0.305) more difficulties in anger management; 20.1% (*r* = 0.201) more aggressive behaviour towards others; 22.0% (*r* = 0.220) more defiant behaviour towards authority figures; and 27.4% (*r* = 0.274) more antisocial behaviour than non-victims. Nevertheless, the model error (probability of the victim group scoring below the mean of the non-victim group) was 33.0% for attention problems, 29.5% for hyperactivity–impulsivity, 26.1% for anger management difficulties, 34.1% for aggression towards others, 32.6% for defiant behaviour towards authority figures, and 28.4% for antisocial behaviour.

The univariate effects for the interaction between victimization and gender (see Table 4) displayed a significant effect on anger management difficulties, aggression behaviours towards others, and challenging behaviours towards authority figures. The standardized mean difference between female (*n* = 168) and male (*n* = 80) victims of sexual harassment was significant (LL of the 95% CI > 0.20) and of small to moderate magnitude (LL 0.20 < *g* < UL 0.50) for aggression towards others, with an effect size greater than 61.03%, and for antisocial behaviour, with an effect size greater than 60.26%.

Although the interaction between the factors victimization and gender was significant in anger control difficulties, the effect for the comparison of interest (female victims vs. male victims) was smaller than small and thus, irrelevant (UL < 0.20). Quantitatively, adolescent victims of sexual harassment reported 19.1% (*r* = 0.191) more aggressive behaviours towards others and 18.2% more antisocial behaviours than adolescent victims. Even so, the model error (probability of the male victim group scoring below the mean of the female victim group) was 34.8% for aggression towards others and 35.6% for antisocial behaviour.

## 4. Discussion

The results showed that around one in four adolescents is a victim of sexual harassment (24.1%, 95% CI [0.215, 0.267]) with a significantly higher prevalence among females than among males. A similar tendency in gender was found in the UK population, but not in prevalence, which was twice more in the UK population; however, in that study, sexual harassment was measured differently than in present study (requiring intentionality of the behaviour/action, periodicity, and chronicity) as the experience of at least one form of sexual harassment (i.e., overestimation of the rate) [51]. The incremental effect on the triviality (net effect) of sexual harassment was 79.2%. Thus, adolescent sexual harassment victimization transcends the trivial in such a way that it has become problem that requires the implementation of prevention programs with the aim of reducing its prevalence to trivial. The implemented programs need to be gender-oriented as the prevalence is higher for females. Nevertheless, the observed prevalence is far from those reported in the previous literature [4,12], which overestimate the prevalence due to errors in the used measures (i.e., the measure does not meet the criteria for sexual harassment).

The results corroborated that sexual harassment victimization brings direct adverse effects on the set of internalizing MHPs, quantified as 33.0% more depressive symptoms (victims reported 33.0% more depressive symptoms than non-victims), 30.9% more anxiety symptoms, 8.5% more social anxiety, 27.9% more somatic complaints, 35.9% more posttraumatic symptoms, and 24.3% more obsessive–compulsive symptoms than non-victims. These findings suggest that school-aged adolescent victims of sexual harassment exhibited more adverse internalizing mental health problems. In summary, the internalizing harm is (multi)comorbid and is not only related, as presumed in the previous literature, to anxious–depressive symptoms. Regarding the judicial context, the verification of harm in posttraumatic symptoms is key to demonstrate a case (charge of the proof) due to that the legal definition of a victim of a crime requires the suffering of harm [19]. Nevertheless, posttraumatic stress disorder (PTSD) is not the correct diagnosis for sexual harassment victimization in forensic evaluation. Differential diagnosis states that Adjustment Disorder is the appropriate (sexual harassment is not listed as a traumatic event) [15]. Moreover, the resulting harm is (multi)comorbid that is linked to severe harm [52,53,54,55,56].

The results also suggested more damage in externalizing MHPs for victims of sexual harassment victimization. These were estimated at an increase of 21.5% in attention problems; 26.1% in manifestations of hyperactivity–impulsivity; 30.5% in anger control; 20.1% in aggressive behaviour towards other people; 22.0% in defiant behaviour towards authority figures; and 27.4% in antisocial behaviour. These results also highlight the need of an intervention in externalizing MHPs associated with sexual harassment victimization with special attention to antisocial behaviours that turn victims into aggressors [30].

Comparatively, the effect was significantly larger for internalizing MHPs (the confidence interval was larger) than for externalizing MHPs, which is in line with the transition at these ages from externalizing (younger age) to internalizing (older age) clinical manifestation.

### 4.1. Limitations

This research is subject to limitations in its generalizability, which should be borne in mind. First, the sampling technique applied has margins of error within which the prevalence estimates may oscillate. Second, an inter-subject measurement design (as opposed to a repeated measures design) was used, which does not allow us to understand the evolution of psychological adjustment in victimized individuals from the perspective of individual development during adolescence. Third, the measurement instruments used were self-report measures, and consequently, they may be subject to response bias on the part of the participants. Both social desirability in responses and denial of harm are suspected. Fourth, the diagnosis of sexual harassment was based on a psychometric measure, which in clinical practice has to be endorsed in a clinical interview. Fifth, the influence of other types of variables not assessed in this research could have mediating effects on the variables under study. Sixth, the measurement instrument was constructed based on previous instruments assuming, and non-contrasting, their real validity. Bearing these limitations in mind, the results obtained are discussed below.

### 4.2. Future Research

With a view to future lines of research, the present study suggests that the relevance of studies aimed at (a) the creation and validation of a measure of sexual harassment with psychometric properties; (b) the specification of the factors associated with peer victimization of sexual harassment at school; (c) the mediating variables of the adverse effects of harassment victimization; and (d) the causes of aggression. The final aim is to provide a better adjustment of prevalence rates, as well as a better understanding of this phenomenon. Hence, these issues should be kept in mind in the educational setting when designing, developing, and implementing prevention and intervention programs to address sexual harassment and, in turn, improve the physical, psychological, and social well-being of young people. The use of Online Photovoice (OPV) methodology in order to understand the prevalence and quantification of the effects is strongly recommended [57].

## Figures and Tables

**Table 1:** Univariate effects on internalizing MHPs for the sexual harassment victimization factor. Between-subject effects.

Internalizing MHPs	*F*	*p*	*g*[95% CI]	1 − β	*M*_VAS_	*M*_N-VAS_	PS_ES_	PIS[95% CI]
Depression	65.67	<0.001	0.70[0.68, 0.72]	1.00	2.86	2.25	0.6879	0.242[0.216, 0.268]
Anxiety	49.89	<0.001	0.65[0.63, 0.67]	1.00	3.48	2.89	0.6772	0.258[0.231, 0.285]
Social anxiety	1.69	<0.001	0.17[0.15, 0.19]	0.255	2.82	2.67	0.5478	0.433[0.403, 0.463]
Somatic complaints	41.86	<0.001	0.58[0.56, 0.60]	1.00	2.91	2.47	0.6591	0.281[0.254, 0.308]
Posttraumatic symptoms	74.20	<0.001	0.77[0.75, 0.79]	1.00	2.68	2.11	0.7054	0.221[0.196, 0.246]
Obsessive–compulsive	29.91	<0.001	0.50[0.45, 0.55]	1.00	2.58	2.22	0.5987	0.308[0.280, 0.336]

Note. *df*(1, 1024); *g*[95% CI]: unbiased Hedges’ *g*[95% confidence interval]; 1 − β: achieved power; *M*_VAS_: mean of the group of victims of sexual harassment; *M*_N-VAS_: mean of the group of non-victims of sexual harassment; PS_ES_: Probability of Superiority of the Effect Size; PIS[95% CI]: Probability of an Inferiority Score[95% confidence interval]; Box’ M = 180.052, *F*(63, 319590.1) = 2.81, *p* < 0.001.

**Table 2:** Univariate effects on internalizing MHPs for the interaction between sexual harassment victimization and gender. Between-subject effects.

Internalizing MHPs	*F*	*p*	*g*[95% CI]	1 − β	*M*_VAS_	*M*_N-VAS_	PS_ES_	PIS[95% CI]
Depression	45.44	<0.001	0.62[0.57, 0.67]	1.00	3.06	2.46	0.6700	0.268[0.213, 0.323]
Anxiety	116.73	<0.001	1.06[1.11, 1.01]	1.00	3.77	2.89	0.7734	0.145[0.101, 0.189]
Social anxiety	28.56	<0.001	0.38[0.33, 0.43]	1.00	2.93	2.58	0.6064	0.352[0.293, 0.411]
Somatic complaints	58.51	<0.001	0.65[0.60, 0.70]	1.00	3.08	2.57	0.6772	0.258[0.204, 0.312]
Posttraumatic symptoms	38.95	<0.001	0.66[0.61, 0.71]	1.00	2.85	2.32	0.6802	0.255[0.201, 0.309]
Obsessive–compulsive	24.08	<0.001	0.55[0.50, 0.60]	1.00	2.72	2.28	0.6517	0.291[0.234, 0.348]

Note. *df*(1, 1024); *g*[95% CI]: unbiased Hedges’ *g*[95% confidence interval]; 1 − β: achieved power; *M*_VAS_: mean of the group of victims of sexual harassment; *M*_N-VAS_: mean of the group of non-victims of sexual harassment; PS_ES_: Probability of Superiority of the Effect Size; PIS[95% CI]: Probability of an Inferiority Score[95% confidence interval]; Box’ M = 180.052, *F*(63, 319590.1) = 2.81, *p* < 0.001.

**Table 3:** Univariate effects on externalizing MHPs for the sexual harassment victimization factor. Between-subject effects.

Externalizing MHPs	*F*	*p*	*g*[95% CI]	1 − β	*M*_VAS_	*M*_N-VAS_	PS_ES_	PIS[95% CI]
Attention problems	39.50	<0.001	0.44[0.39, 0.49]	1.00	2.94	2.57	0.6217	0.330[0.301, 0.359]
Hyperactivity–impulsivity	58.02	<0.001	0.54[0.49, 0.59]	1.00	2.57	2.19	0.6480	0.295[0.267, 0.323]
Anger control	68.62	<0.001	0.64[0.59, 0.69]	1.00	2.51	2.03	0.6736	0.261[0.234, 0.288]
Aggression	48.20	<0.001	0.41[0.36, 0.46]	1.00	1.53	1.34	0.6141	0.341[0.312, 0.370]
Defiant behaviour	45.89	<0.001	0.45[0.40, 0.50]	1.00	1.87	1.57	0.6255	0.326[0.297, 0.355]
Antisocial behaviour	81.55	<0.001	0.57[0.52, 0.57]	1.00	1.46	1.24	0.6554	0.284[0.256, 0.312]

Note. *df*(1, 1024); *g*[95% CI]: unbiased Hedges’ *g*[95% confidence interval]; 1 − β: achieved power; *M*_VAS_: mean of the group of victims of sexual harassment; *M*_N-VAS_: mean of the group of non-victims of sexual harassment; PS_ES_: Probability of Superiority of the Effect Size; PIS[95% CI]: Probability of an Inferiority Score[95% confidence interval]; Box’ M = 338.343, *F*(63, 318590.1) = 5.28, *p* < 0.001.

**Table 4:** Univariate effects on externalizing MHPs for the interaction between sexual harassment victimization and gender. Between-subject effects.

Externalizing MHPs	*F*	*p*	*g*[95% CI]	1 − β	*M*_HVA_	*M*_MVA_	PS_ES_	PIS[95% CI]
Attention problems	0.97	0.379	0.17[0.12, 0.22]	0.220	3.05	2.89	0.5478	0.433[0.371, 0.495]
Hyperactivity–impulsivity	1.60	0.203	0.19[0.14, 0.24]	0.339	2.67	2.52	0.5517	0.425[0.363, 0.487]
Anger control	4.96	0.007	−0.12[−0.17, −0.07]	0.811	2.44	2.55	0.5319	0.452[0.390, 0.514]
Aggression	14.54	<0.001	0.39[0.34, 0.44]	0.999	1.68	1.46	0.6103	0.348[0.289, 0.407]
Defiant behaviour	2.55	0.079	0.20[0.15, 0.25]	0.510	1.98	1.82	0.5557	0.421[0.360, 0.482]
Antisocial behaviour	10.25	<0.001	0.37[0.32, 0.42]	0.987	1.58	1.40	0.6026	0.356[0.296, 0.416]

Note. *df*(1, 1024); *g*[95% CI]: unbiased Hedges’ *g*[95% confidence interval]; 1 − β: achieved power; *M*_VAS_: mean of the group of victims of sexual harassment; *M*_N-VAS_: mean of the group of non-victims of sexual harassment; PS_ES_: Probability of Superiority of the Effect Size; PIS[95% CI]: Probability of an Inferiority Score[95% confidence interval]; Box’ M = 338.343, *F*(63, 318590.1) = 5.28, *p* < 0.001.

## Data Availability

The data presented in this study are available on request from the corresponding author. The data are not publicly available due to legal restrictions of data from underage participants.

## Appendix A. Inventario de Conductas y Estrategias de Acoso Sexual en la Infancia y en la Adolescencia [Inventory of Sexual Harassment Behaviors and Strategies in Childhood and Adolescence]

Below there are some behaviors that may be directed at you. Please answer if you have happened to yourself, in an unwanted way, by a student or students. Remember that it must always be unwanted. Answer honestly.

**Table A1:** Sexual Harassment Behaviors and Strategies.

	0 = Never or rarely happens to me 1 = Once a month 2 = Two or three times a month 3 = Once a week 4 = Several times a week.					If you answered “once a week” or “several times a week”, please indicate how long you have been subjected to this situation.
1. You have made comments, mockery or sexual gestures towards you on your Social Network profile, through a messaging platform (WhatsApp, Telegram,...) or any other medium.	0	1	2	3	4	□ Until 1 month □ Until 3 months □ Until 6 months □ Until 1 year more or
2. You have been shown, given or left images, photographs or comments of a sexual nature.	0	1	2	3	4	□ Until 1 month □ Until 3 months □ Until 6 months □ Until 1 year more or
3. You have written you sexual messages or have shown by yourself sexual drawings.	0	1	2	3	4	□ Until 1 month □ Until 3 months □ Until 6 months □ Until 1 year more or
4. You speak about sex with you face to face, on the Internet or other (unwanted) media.	0	1	2	3	4	□ Until 1 month □ Until 3 months □ Until 6 months □ Until 1 year more or
5. You have mocked or spread false rumours about your sexual behavior on your social media profile, through a messaging platform (WhatsApp, Telegram,...) or other means.	0	1	2	3	4	□ Until 1 month □ Until 3 months □ Until 6 months □ Until 1 year more or
6. You have called you a faggot, lesbian, whore, homosexual, slut, bitch, etc. on your social network profile, through a messaging platform (WhatsApp, Telegram,...) or other means.	0	1	2	3	4	□ Until 1 month □ Until 3 months □ Until 6 months □ Until 1 year more or
7. You have been teased or made fun of about private parts of your body (e.g., penis, vulva, bum or breasts) or about sex with you.	0	1	2	3	4	□ Until 1 month □ Until 3 months □ Until 6 months □ Until 1 year more or
8. You suggested or asked you to send pictures of a naked part of your body.	0	1	2	3	4	□ Until 1 month □ Until 3 months □ Until 6 months □ Until 1 year more or
9. You have been sent or shown a personal photo of a provocative nature or showing a sexual body part.	0	1	2	3	4	□ Until 1 month □ Until 3 months □ Until 6 months □ Until 1 year more or
10. You made sexual comments, jokes, moves or glances at you.	0	1	2	3	4	□ Until 1 month □ Until 3 months □ Until 6 months □ Until 1 year more or
11. Intentionally brushed against you on a sexual manner.	0	1	2	3	4	□ Until 1 month □ Until 3 months □ Until 6 months □ Until 1 year more or
12. You have been pushed and/or pinned against a wall or similar with sexual intent.	0	1	2	3	4	□ Until 1 month □ Until 3 months □ Until 6 months □ Until 1 year more or
13. Your private parts (e.g., penis, vagina, buttocks or breasts) have been touched.	0	1	2	3	4	□ Until 1 month □ Until 3 months □ Until 6 months □ Until 1 year more or
14. Your clothes have been taken off or pulled down in front of you on a sexual manner.	0	1	2	3	4	□ Until 1 month □ Until 3 months □ Until 6 months □ Until 1 year more or
15. You have been blocked, cornered or cornered in a sexual way.	0	1	2	3	4	□ Until 1 month □ Until 3 months □ Until 6 months □ Until 1 year more or
16. You have been forcibly kissed or hugged.	0	1	2	3	4	□ Until 1 month □ Until 3 months □ Until 6 months □ Until 1 year more or
17. You have been touched, grabbed or pinched in a sexual way.	0	1	2	3	4	□ Until 1 month □ Until 3 months □ Until 6 months □ Until 1 year more or
18. You have shown, given or left you sexual pictures, images, messages or comments.	0	1	2	3	4	□ Until 1 month □ Until 3 months □ Until 6 months □ Until 1 year more or
19. You have had your clothes taken off.	0	1	2	3	4	□ Until 1 month □ Until 3 months □ Until 6 months □ Until 1 year more or

Note. This is the English translation of the original Spanish version.
